# Whole-Genome Sequences of SARS-CoV-2 Isolates from Ethiopian Patients

**DOI:** 10.1128/MRA.00721-21

**Published:** 2021-09-23

**Authors:** Dawit Hailu Alemayehu, Bethlehem Adnew, Fekadu Alemu, Dessalegn Abeje Tefera, Tamrayehu Seyoum, Getachew Tesfaye Beyene, Tesfaye Gelanew, Abel Abera Negash, Markos Abebe, Adane Mihret, Abebe Genetu Bayih, Alemseged Abdissa, Andargachew Mulu

**Affiliations:** a Armauer Hansen Research Institute, Addis Ababa, Ethiopia; DOE Joint Genome Institute

## Abstract

Three complete severe acute respiratory syndrome coronavirus 2 (SARS-CoV-2) genomes from Ethiopian patients were compared with deposited global genomes. Two genomes belonged to genetic group 20A/B.1/GH, and the other belonged to genetic group 20A/B.1.480/GH. Enhancing genomic capacity is important to investigate the transmission and to monitor the evolution and mutational patterns of SARS-CoV-2 in this country.

## ANNOUNCEMENT

The severe acute respiratory syndrome coronavirus 2 (SARS-CoV-2) that emerged in Wuhan, China, is an RNA virus that belongs to the genus *Betacoronavirus*, in the family *Coronaviridae* (1). Like most RNA viruses, SARS-CoV-2 is expected to display a relatively high rate of genetic mutations, which may influence viral transmission and pathogenesis, enable escape from host defenses, and negatively affect the efficacy of vaccines and molecular diagnostic tools (2). Thus, enhancing genomic capacity is important to investigate the transmission and to monitor the evolution and mutational patterns of SARS-CoV-2 in this country.

Here, we report three SARS-CoV-2 genome sequences using Illumina NextSeq sequencing technology. The protocol was ethically approved by the ALERT/AHRI Research Ethics Committee. Nasopharyngeal swab samples were collected from subjects with suspected SARS-CoV-2 following routine surveillance and diagnostic procedures. The first two samples (GenBank accession numbers MZ172407 and MZ172408) were collected from a hospital setting, and the last one (GenBank accession number MZ172409) was collected from a health center. Nucleic acid was extracted using a Da An Gene extraction kit (catalog number DA0591) following the manufacturer’s protocol. The extracted RNA was reverse transcribed and SARS-CoV-2 was detected using the BGI real-time fluorescent reverse transcription (RT)-PCR kit (catalog number MFG030010). Positive RNA samples were selected for sequencing based on their threshold cycle (*C_T_*) values (*C_T_* values of <24). The RNA was concentrated using SPRI magnetic beads, and reverse-transcribed RNA was sequenced using the shotgun metagenomic workflow outlined by Illumina (3). In short, 200 to 450 ng of input RNA was subjected to ribodepletion, fragmentation, first- and second-strand cDNA synthesis, adenylation, adapter ligation, and amplification, according to the TruSeq stranded total RNA protocol. The prepared libraries were loaded on the NextSeq 500 system for a paired-end 2 × 76-bp sequencing run. The base call (BCL) files from the NextSeq 500 system were demultiplexed and converted to FASTQ files using Illumina bcl2fastq2 software v2.20. Quality-checked paired-end FASTQ files (4) were trimmed using Trimmomatic v0.36 (5). Taxonomic classification was performed using Kraken2 (6), and the host reads were removed using Bowtie2 (7) and SAMtools (8) with the human reference genome (GRCh38) (ftp://ftp.ccb.jhu.edu/pub/data/bowtie_indexes) to yield unmapped reads. The reads with the host reads removed were aligned to the complete genome of SARS-CoV-2 Wuhan-Hu-1 (GenBank accession number NC_045512.2) using BWA (9), and SAMtools was used for intermediate file conversion and summary. Ivar consensus sequences were used as genome sequences. Variants were called using Snippy (https://github.com/tseemann/snippy) and Nextclade. Local Nextstrain/Nextclade v0.13.0 was also implemented for clade assignment and variant annotation. The phylogenetic tree was generated with Nextstrain/Augur using its default subsampling scheme and focusing on country Ethiopia, region Africa, where 1,960 samples were subsampled between December 2019 and February 2021; the tree was visualized using the Nextstrain/Auspice tool. Lineage assignments were made using the Phylogenetic Assignment of Named Global Outbreak Lineages (Pangolin) v1.07 tool (https://github.com/hCoV-2019/pangolin) and clades from GISAID (https://www.gisaid.org). All tools were run with default parameters unless otherwise specified. There is 99.68 to 99.92% sequence identity using BLAST between the full genome sequences of the isolates and the reference strain at the nucleotide level and 99.94% identity at the amino acid level. All three isolates have 99.97 to 100% coverage, with 100% coverage of the coding region. The genome sizes were 29,860, 29,856, and 29,871 bp, with GC contents of 53%, 51%, and 49%, for isolates MZ172407, MZ172408, and MZ172409, respectively. Similarly, the average coverage depths were 2,56.7× (range, 1× to 3,183×), 23.8× (range, 1× to 1,110×), and 1,288.3× (range, 4× to 8,002×) for the isolates MZ172407, MZ172408, and MZ172409, respectively.

**FIG 1:**
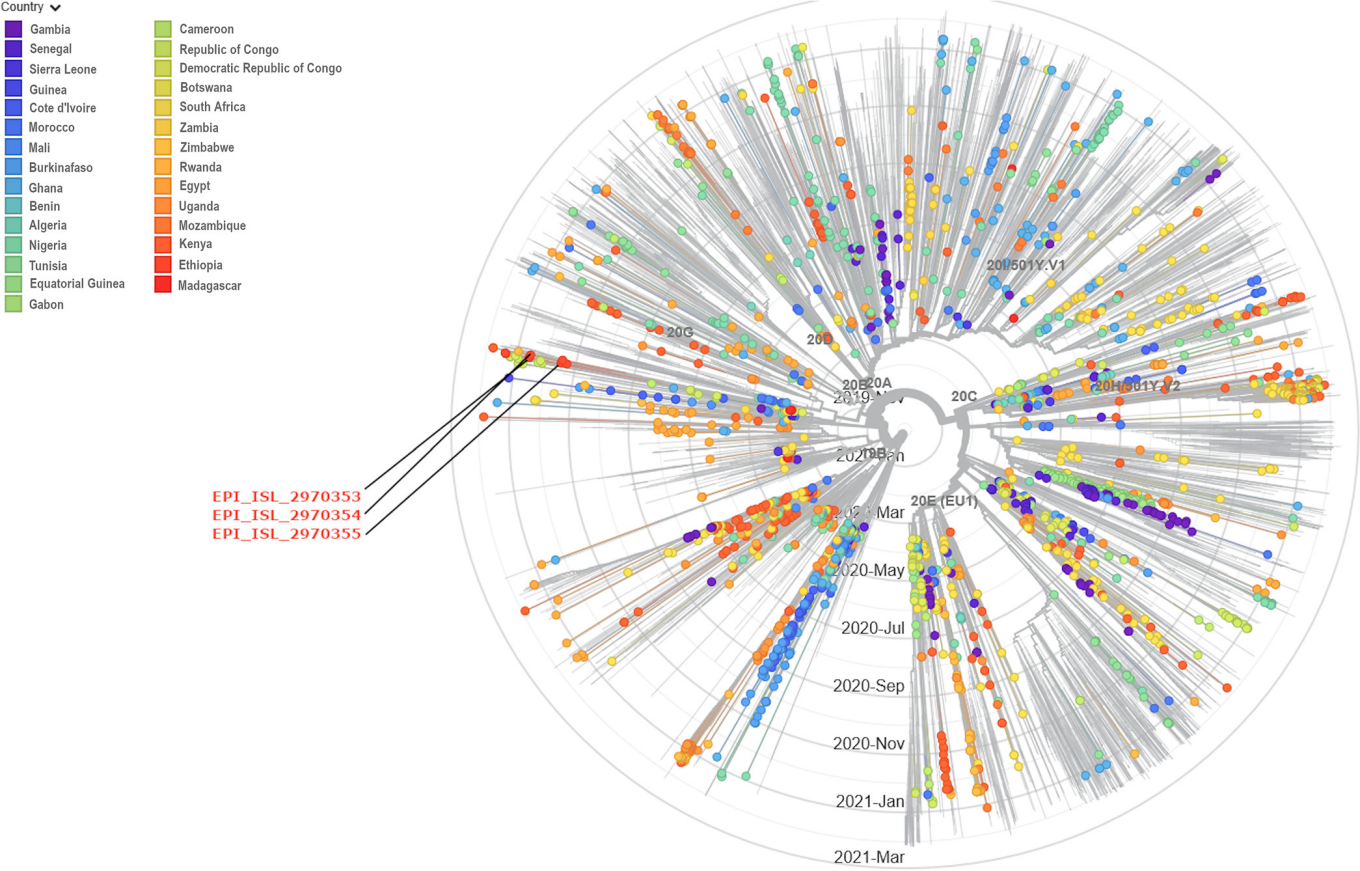
Phylogenetic analysis of representative SARS-CoV-2 genome sequences, including the three current isolates. Available genomes were retrieved from GISAID (https://www.gisaid.org) in January 2021. Sequences with low quality (i.e., ambiguous bases) were discarded. The figure was created using Nextstrain.

Phylogenomic analysis showed that two of the detected SARS-CoV-2 isolates (isolates MZ172408 and MZ172409) belonged to lineage B.1 of the Pangolin lineage, sharing the most common recent ancestor with viruses detected in Germany (Fig. 1). One of the isolates (isolate MZ172407) was found to belong to lineage B.1.480. According to Nextstrain (10), the phylogenetic tree revealed that all of the isolates belonged to Nextstrain clade 20A and GISAID clade GH.

Mutations among the three SARS-CoV-2 strains were identified throughout the whole genome, with reference to the SARS-CoV-2 Wuhan strain (GenBank accession number NC_045512.2), and marked nucleotide differences in some positions were found, as shown in Table 1. In general, several synonymous and nonsynonymous mutations with pyrimidine exchanges (C to T or T to C) (55%) were observed in all three genomes (Table 1). Currently, we are sequencing more genomes to further investigate the transmission and to monitor the evolution and mutational patterns of SARS-CoV-2 in this country.

**TABLE 1:** Alterations of the SARS-CoV-2 genome

Amino acid position^*a*^	Base					Gene^*b*^	Protein^*c*^	Amino acid substitution	Mutation type
	Reference	Alternative	Isolate MZ172407	Isolate MZ172408	Isolate MZ172409				
140	C	T	C	C	T	5′-UTR	NA	NA	Noncoding
241	C	T	T	T	T	5′-UTR	NA	NA	Noncoding
875	C	T	T	C	C	ORF1ab	ORF1ab polyprotein/NSP2	L204F	Missense
936	C	T	C	C	T	ORF1ab		T224I	Missense
2300	T	C	T	C	C	ORF1a	ORF1ab polyprotein/NSP2	F679L	Missense
2416	C	T	T	T	T	ORF1ab	NSP2		Missense
2445	C	T	T	C	T	ORF1ab	NSP2	T727I	Missense
3037	C	T	T	T	T	ORF1ab	NSP3		Missense
3643	A	G	T	T	G	ORF1ab			Synonymous
4071	C	T	T	C	C	ORF1ab	NSP3	T1269I	Synonymous
4280	G	A	A	G	G	ORF1ab	ORF1ab polyprotein/NSP2	V1339I	Missense
7534	T	C	C	T	T	ORF1ab	ORF1ab polyprotein/NSP2		Synonymous
9724	C	T	T	C	C	ORF1ab	ORF1ab polyprotein/NSP2		Synonymous
10904	A	G	G	A	A	ORF1ab	ORF1ab polyprotein/NSP2	S3547G	Missense
11758	C	T	C	T	C	ORF1ab	ORF1ab polyprotein/NSP2		Synonymous
12076	C	T	T	C	C	ORF1ab	ORF1ab polyprotein/NSP2		Synonymous
14022	C	T	T	C	C	ORF1ab	ORF1ab polyprotein/NSP2		Synonymous
14407^*d*^	C	T	C	C	T	ORF1ab			Synonymous
14408	C	T	T	T	T	ORF1ab	ORF1ab polyprotein/NSP12	P314L	Missense
14925	C	T	C	C	T	ORF1ab			Synonymous
15384	G	T	T	G	G	ORF1ab	ORF1ab polyprotein/NSP12	L639F	Missense
16269	G	A	A	G	G	ORF1ab	ORF1ab polyprotein		Missense
16647	G	T	G	T	T	ORF1ab			Synonymous
21619	A	T	T	A	A	S	Spike protein		Missense
21721	C	T	C	T	C	S	Spike protein		Missense
21796	G	T	G	G	T	S	Spike protein		Missense
21800	G	T	G	G	T	S	Spike protein	D80Y	
23063	A	T	T	A	A	S	Spike protein	N501Y	Synonymous
23403	A	G	G	G	G	S	Spike protein	D614G	Synonymous
24070	A	C	A	A	C	S	Spike protein	Q836H	Missense
25249	G	T	T	G	G	S	Spike protein	M1229I	Missense
25563	G	T	T	T	T	ORF3a		Q57H	Missense
25844	G	T	T	G	G	ORF3a		T151I	Synonymous
25904	C	T	C	T	C	ORF3a	ORF3a protein	S171L	Missense
26416	G	C	C	G	G	E	E protein	V58L	Synonymous
27484	T	C	C	T	T	ORF7a	ORF7a protein		Synonymous
27546	T	C	T	C	T	ORF6	ORF 6 protein		
27667	G	A	G	G	A	ORf7a		E92K	Upstream
28854	C	T	T	C	C	N	N protein	S194L	Synonymous
28869	C	T	C	T	T	N	N protein	P199L	Missense
29550	C	T	C	T	T	N	N protein	NA	
29702	G	A	A	G	G	3′-UTR			NA

a Variants were called using Snippy (https://github.com/tseemann/snippy) and Nextclade.

b UTR, untranslated region; ORF, open reading frame.

c NA, not applicable; NSP, nonstructural protein.

d Multiple-nucleotide polymorphism (CC to TT).

### Data availability.

The coding-complete sequences were deposited in GenBank with accession numbers MZ172407, MZ172408, and MZ172409 and SRA accession numbers SAMN20692030, SAMN20692031, and SAMN20692032 and in GISAID (https://www.gisaid.org) with accession numbers EPI_ISL_2970353, EPI_ISL_2970354, and EPI_ISL_2970355 for Ethiopia/AHRI-01/2020, Ethiopia/AHRI-02/2020, and Ethiopia/AHRI-03/2020, respectively.

## References

[1] Wu F, Zhao S, Yu B, Chen Y-M, Wang W, Song Z-G, Hu Y, Tao Z-W, Tian J-H, Pei Y-Y, Yuan M-L, Zhang Y-L, Dai F-H, Liu Y, Wang Q-M, Zheng J-J, Xu L, Holmes EC, Zhang Y-Z. 2020. A new coronavirus associated with human respiratory disease in China. Nature 579:265–269. doi:10.1038/s41586-020-2008-3. 32015508 PMC7094943

[2] Miao M, Clercq ED, Li G. 2021. Genetic diversity of SARS-CoV-2 over a one-year period of the COVID-19 pandemic: a global perspective. Biomedicines 9:412. doi:10.3390/biomedicines9040412. 33920487 PMC8069977

[3] Illumina. 2020. Comprehensive workflow for detecting coronavirus using Illumina benchtop systems. Illumina, San Diego, CA. https://emea.illumina.com/content/dam/illumina-marketing/documents/products/appnotes/ngs-coronavirus-app-note-1270-2020-001.pdf.

[4] Andrews S. 2010. FastQC: a quality control tool for high throughput sequence data. http://www.bioinformatics.babraham.ac.uk/projects/fastqc.

[5] Bolger AM, Lohse M, Usadel B. 2014. Trimmomatic: a flexible trimmer for Illumina sequence data. Bioinformatics 30:2114–2120. doi:10.1093/bioinformatics/btu170. 24695404 PMC4103590

[6] Wood DE, Lu J, Langmead B. 2019. Improved metagenomic analysis with Kraken 2. Genome Biol 20:257. doi:10.1186/s13059-019-1891-0. 31779668 PMC6883579

[7] Langmead B, Salzberg SL. 2012. Fast gapped-read alignment with Bowtie 2. Nat Methods 9:357–359. doi:10.1038/nmeth.1923. 22388286 PMC3322381

[8] Li H, Handsaker B, Wysoker A, Fennell T, Ruan J, Homer N, Marth G, Abecasis G, Durbin R, 1000 Genome Project Data Processing Subgroup. 2009. The Sequence Alignment/Map format and SAMtools. Bioinformatics 25:2078–2079. doi:10.1093/bioinformatics/btp352. 19505943 PMC2723002

[9] Li H, Durbin R. 2009. Fast and accurate short read alignment with Burrows-Wheeler transform. Bioinformatics 25:1754–1760. doi:10.1093/bioinformatics/btp324. 19451168 PMC2705234

[10] Hadfield J, Megill C, Bell SM, Huddleston J, Potter B, Callender C, Sagulenko P, Bedford T, Neher RA. 2018. Nextstrain: real-time tracking of pathogen evolution. Bioinformatics 34:4121–4123. doi:10.1093/bioinformatics/bty407. 29790939 PMC6247931

